# Promotion of Family Routines and Positive Parent-Child Interactions for Obesity Prevention: Protocol for the 3 Pillars Study Randomized Controlled Trial

**DOI:** 10.2196/12792

**Published:** 2019-04-02

**Authors:** Samantha Marsh, Sarah Gerritsen, Rachael Taylor, Barbara Galland, Varsha Parag, Ralph Maddison

**Affiliations:** 1 National Institute for Health Innovation School of Population Health University of Auckland Auckland New Zealand; 2 School of Population Health University of Auckland Auckland New Zealand; 3 Department of Medicine University of Otago Dunedin New Zealand; 4 Department of Women’s and Children’s Health University of Otago Dunedin New Zealand

**Keywords:** screen time, family routines, parent-child relations, child, preschool, randomized controlled trial, health behavior, pediatric obesity, sleep, parenting, New Zealand

## Abstract

**Background:**

Childhood obesity is a challenging public health issue, with 30% of children aged 2 to 4 years classified as being overweight or obese in New Zealand. This is concerning, given that up to 90% of obese 3-year-old children are overweight or obese by the time they reach adolescence. Interventions that target this age range often fail to demonstrate long-term effectiveness and primarily focus on traditional weight-related behaviors, including diet and physical activity. However, research suggests that targeting nontraditional weight-related behaviors, such as sleep, screen time, and family meals, may be a more effective approach in this age group, given the immense challenges in changing traditional weight-related behaviors in the long term.

**Objective:**

The aim of the proposed study was to develop and pilot the 3 Pillars Study (3PS), a 6-week program for parents of New Zealand toddlers and preschoolers aged 2 to 4 years to promote positive parent-child interactions during 3 family routines, specifically adequate sleep, regular family meals, and restricted screen time.

**Methods:**

Screen time at the end of the 6-week program is the primary endpoint. The effects of the program on screen time, frequency of family meals, parent feeding practices, diet quality, and sleep duration will be piloted using a randomized controlled trial, with outcomes compared between the active intervention group and a wait-list control group at 6 weeks (at the end of the program) and 12 weeks (at final follow-up). We aim to recruit 50 participants (25 per arm). Eligibility criteria include parents of children aged 2 to 4 years who are currently exceeding screen use recommendations (ie, greater than 1 hour of screen time per day). The 3PS program involves a half-day workshop, run by a community worker trained to deliver the program content, and 6-week access to a study website that contains in-depth information about the program. All participants will also receive a study pack, which includes resources to encourage engagement in the 3 family routines promoted by the program. Study data will be collected in REDCap. All statistical analyses will be performed using SAS version 9.4 and have been specified a priori in a statistical analysis plan prepared by the study statistician.

**Results:**

Trial recruitment opened in July 2018. Final follow-up was completed in December 2018, with trial findings expected to be available in early 2019.

**Conclusions:**

Findings from this pilot study will provide relevant data to inform the design of a larger effectiveness study of the 3PS program.

**Trial Registration:**

Australian New Zealand Clinical Trials Register ACTRN12618000823279; https://www.anzctr.org. au/Trial/Registration/TrialReview.aspx?id=375004 (Archived by WebCite at http://www.webcitation.org/773CALeTK)

**International Registered Report Identifier (IRRID):**

DERR1-10.2196/12792

## Introduction

### Background

Childhood obesity remains one of the most pervasive and challenging public health issues, with 30% of children aged 2 to 4 years classified as being overweight or obese in New Zealand [1]. Once a child has developed obesity, it is difficult to reverse, with weight status in the first years of life a strong predictor for adult obesity [2]. In particular, the interval between 2 and 6 years has been identified as the earliest and most critical period of growth with respect to the future risk of obesity in adolescence [3] or adulthood [4]. Of concern is recent evidence suggesting that almost 90% of children who are obese at the age of 3 years are classified as overweight or obese as an adolescent [3]. Given the intractable nature of obesity, there has been a shift in recent years toward focusing on early prevention of obesity [5].

Although a number of interventions targeting obesity prevention in toddlers and preschoolers have been undertaken, results have been mixed [6,7], with a 2011 systematic review finding no evidence for effectiveness with respect to body weight outcomes [8]. Most studies have focused on traditional weight-related behaviors, including diet, physical activity, and sedentary behaviors. However, recent research has suggested the need to consider other nontraditional weight-related behaviors [9], which seems appropriate given the immense challenges in changing these behaviors in the long term [10,11].

For example, the Prevention of Overweight in Infancy study found that children randomized to receive additional support for more traditional weight-related behaviors, including breastfeeding, healthy eating, and physical activity during the first 2 years of life, had significantly higher body mass index (BMI) z scores compared with controls at age 5 years (adjusted difference: 0.25; 95% CI 0.04 to 0.47) [12]. Perhaps even more interesting was the observation that those randomized to receive education and support for sleep, either alone or in combination with support for the traditional weight-related behaviors, had significantly lower BMI z scores at both age 3.5 years (−0.24; 95% CI −0.38 to −0.10) and 5 years (−0.23; 95% CI −0.38 to −0.07), compared with children who did not receive the sleep intervention [12].

There are now a growing number of experts calling for a shift to identify and focus on these nontraditional weight-related behaviors, including adequate night-time sleep, regular participation in family meals, and limiting screen time [5,12-20], which are frequently referred to as family routines. In fact, these family routines are showing promise with respect to obesity prevention in young children. In the United States, preschool children from households that regularly engaged in these 3 family routines had approximately 40% lower prevalence of obesity compared with those exposed to none of the routines [13]. Similar findings have been reported in New Zealand, where 3 primary distinctions between low socioeconomic status (SES) Pacific families with healthy-weight children and low SES Pacific families with overweight children were identified: engagement in regular family meals, presence of food-related rules at home, and limitations on screen time [20]. Furthermore, promoting family routines in an intervention has been shown to increase engagement in healthy body weight–related behaviors and reduce BMI, in low-income minority families with young children [15].

It is likely that family routines actually represent the broader constructs of family functioning and family organization [21,22]. With respect to family functioning, ongoing participation in a routine may be an indication that family members experience the routine to be a positive and rewarding activity. In this case, other members of the family, rather than just an individual parent, may work to ensure regular engagement in the routine. Alternatively, if individual members of the family find a routine to be stressful, for example, if sitting down to eat dinner together often results in parent-child conflict, then it is less likely that they will actively try to overcome barriers to making the routine work on a regular basis.

Second, it makes sense pragmatically that organized families will simply be better placed to arrange their time and resources in such a way that it facilitates both structuring of routines in the first instance and then ongoing participation in them. For example, children from disorganized families are significantly more likely to demonstrate sleep problems, and it is proposed that the disorganization these families experience interferes with their ability to engage in a regular bedtime routine, which is known to support healthy sleep in children [23].

The approach of targeting family routines, such as sleep and family meals, rather than traditional weight-management behaviors, such as diet and physical activity, may have a number of benefits. First, parents do not need to perceive their child to be at risk of being overweight or feel criticized for their parenting behaviors [13]. This may be particularly important for New Zealand families, where negative cultural discourses around *skinny* children exist [24] and where findings from a longitudinal study recently found that 73% of mothers with an overweight or obese child reported their child to be normal weight [25]. Second, family routines appear to offer nonweight-related benefits, including improved resiliency, cognitive skills, self-regulation, school readiness, behavior, and psychosocial well-being [26-29].

Third, although family routines are directly modifiable by parents, more traditional weight-management behaviors, such as what and how much a child eats, are also affected by a child’s individual characteristics, such as temperament [30] and self-regulation [31]. As a result, although these traditional behaviors may be more difficult for the parent to modify directly, changing family routines around meals, bedtime, and screen use may represent a pathway to influencing these behaviors indirectly. Finally, lack of family routines, as represented by family disorganization, appears to be overrepresented in socioeconomically disadvantaged families [32], with children from these families appearing to also be more susceptible to the adverse effects of disorganization [33]. Indeed, research suggests that family disorganization may play a mediating role in the relationship between SES and child outcomes [33-35]. Although we currently do not know if household chaos can readily be reduced in disadvantaged families, although preliminary evidence suggests it can [15], it may represent a more actionable and immediate target for improving child outcomes, particularly with respect to childhood obesity.

It may also be valuable to consider the importance of promoting positive parent-child interactions during family routines associated with obesity prevention. It is possible that families eating together at mealtimes may only protect against childhood obesity because of positive parent-child interactions [36,37]. Focusing primarily on the association between family meal frequency and body weight, with no consideration for parent-child interactions at family meals, may fail to capture the complexity of the family meal experience. Negative family meal experiences and food-related parenting practices such as coercive feeding may actually increase the risk for unhealthy dietary choices in children and result in an increased risk for obesity [36-38]. Similarly, for sleep, the quality of parent-child interactions appears to be positively associated with night-time sleep in preschool-aged children [39], and positive, connecting routines before bedtime not only have been shown to reduce the number of bedtime tantrums in toddlers and preschoolers but also significantly improved marital satisfaction [40].

The parent-child relationship appears important with respect to obesity prevention, with poorer quality of interactions during playtime interactions, lower maternal sensitivity, and insecure attachment, all associated with obesity risk [41-44]. Researchers, therefore, propose that obesity interventions should include a component that promotes positive parent-child interactions [43,45,46]. Specifically, a 2011 systematic review that investigated the role of parent-child interactions and obesity prevention identified the importance of extending the current model of parent-child interactions [46]. Current models typically focus on unidirectional aspects of parenting, such as parenting practices and parenting styles; however, moving to a bidirectional model would help underscore the importance of dyadic mutuality, defined as the existence of warm, *mutually* responsive, and synchronized interactions between the parent and child [47-49]. This approach acknowledges that the development and maintenance of health-related behaviors is a bidirectional process between the parent and child, whereby exchanges during health-promoting routines, such as family meals and bedtime, need to be mutually rewarding and positive to encourage ongoing engagement in them [46,50].

### Objectives

The aim of the proposed study was to develop and pilot the 3 Pillars Study (3PS), a 6-week program for parents of New Zealand toddlers and preschoolers aged 2 to 4 years to promote positive bidirectional interactions between the parent and child during 3 family routines shown to protect against childhood obesity. Specifically, the program targets adequate night-time sleep, engaging in regular family meals, and restricting screen time. These 3 routines represent the *3 Pillars*. The effects of the program on screen time, frequency of family meals, parent feeding practices, diet quality, and sleep duration will be assessed using a randomized controlled trial (RCT), with outcomes compared between the active intervention group and a wait-list control group at 6 weeks (at the end of the program) and 12 weeks (at final follow-up).

## Methods

### Study Design

A 2-arm, 6-week pilot RCT, with final follow-up at 12 weeks (ie, 6 weeks after the end of the 6-week program), will be conducted to assess 3PS. Intervention participants will attend 1 half-day workshop and have access to a study website with supplementary information for a period of 6 weeks. Both the intervention group and wait-list control group will undergo study measures at baseline, 6 weeks, and 12 weeks. After the final data collection at 12 weeks, the control group will be offered the intervention. The study has been approved by the University Auckland Human Participants Ethics Committee (UAHPEC; reference 021311) and is registered with the Australian New Zealand Clinical Trial Registry (ACTRN12618000823279).

### Participants

A total of 50 participants, 25 in the intervention group and 25 in the wait-list control group, will be recruited to the free 3PS program through social media (ie, targeted Facebook advertising), playgroups, and word of mouth. Participants will be eligible for inclusion if they are the parent or primary caregiver of a child aged 2 to 4 years, if they are aged at least 18 years, and if their child exceeds recommendations for screen use, that is >1 hour per day for this age group, as reported by the parent. Participants will be required to live in Auckland, have internet access, be available to attend 1 half-day workshop, and be able to provide electronic informed consent and speak and read English. Due to the exploratory nature of the intervention, parents of children with serious physical or mental illness or known developmental problems will not be eligible to participate.

### Setting

This study will be conducted in Auckland, New Zealand. The 3PS workshop will take place at the University of Auckland, and participants will be able to access the study website wherever they have access to the internet.

### Recruitment

**Figure 1:**
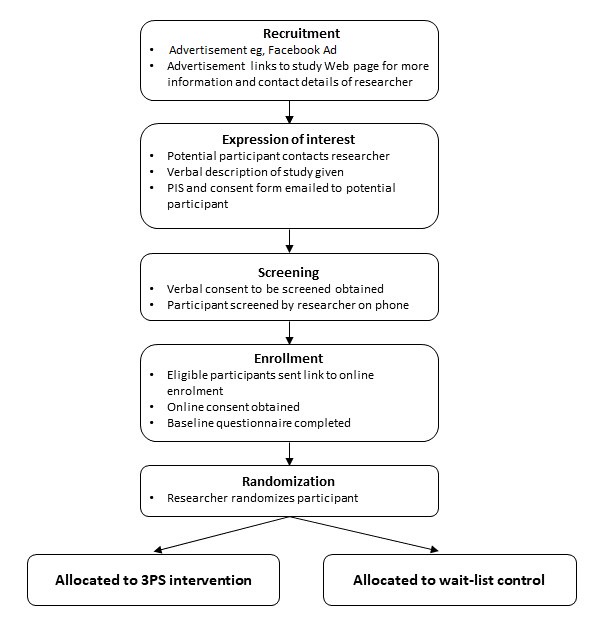
Flowchart illustrating the enrollment and randomization process. 3PS: 3 Pillars Study.

Figure 1 illustrates the recruitment process. A Facebook advertisement will be used for recruitment purposes, with the advertisement offering help to parents having trouble with their child’s screen time, meals, or sleep. This method has been used successfully in the past by the research team for recruiting parents of young children. The advertisement will link to the University of Auckland Faculty of Medical and Health Sciences research study recruitment page, where participants will be given more information about the study and provided with the contact details for the research assistant involved in the study. When potential participants contact the research assistant, an explanation of the study will be given verbally, and they will be sent the Participant Information Sheet and Consent Form via email. If the potential participant expresses their interest in participating, then the research assistant will ask for verbal consent to screen them. Upon agreement, they will be screened by the research assistant over the phone and, if eligible, sent a link to the Web-based baseline questionnaire. Before completing the baseline questionnaire, they will be asked to provide electronic consent to participate in the study. Once they agree to participate, they will be able to complete the baseline questionnaire.

After the baseline questionnaire is complete, the research assistant will randomize the participant to either the intervention group or the wait-list control and inform them of their randomization group. Completion of the baseline questionnaire and randomization will take place during the 2 weeks before the first workshop.

### Randomization

The research assistant will use sequentially numbered, opaque, sealed envelopes to randomize participants. Participants will be allocated to 1 of the 2 groups in a 1:1 ratio using block randomization with varying block sizes of 2 and 4. The randomization sequence will be generated by the study statistician, who will also prepare and seal the envelopes.

### Intervention Development

The 3PS program is designed to promote mutually responsive, positive parent-child interactions and help primary caregivers, herein referred to as parents, of children aged 2 to 4 years to engage in 3 family routines shown to be protective against childhood obesity: adequate night-time sleep, restricted screen time, and regular participation in family meals. The content development process took place over an 18-month period and involved 2 sets of community focus groups, face-to-face parent interviews, and a series of end-user panels with parents. Recruitment criteria used for the formative work was the same as that described above for the pilot study.

Community focus groups (5 focus groups; n=26) and one-on-one interviews (n=8) with parents of 2- to 4-year olds were conducted to establish perceived barriers to engaging in healthy weight-related behavior recommendations in the context of a young family, in addition to establishing the preferred delivery method for a parent intervention. Feedback from the focus groups revealed that one of the biggest barriers to engaging in health-promoting activities was being too busy, either due to family commitments; rushing between activities and chores; or having 2 parents who worked outside the home. In particular, parents said they wanted help with managing the *busyness* of daily life and support for dealing with their children’s challenging behaviors around bedtimes and mealtimes. Furthermore, they indicated that any intervention should be framed in such a way as to avoid feelings of guilt.

When asked how they deal with busyness, parents frequently reported using screens to distract their children while they got things done; yet, at the same time described harboring feelings of guilt about the amount of time their child spent on screens. These conflicting feelings around child screen use were further highlighted when parents conveyed negative responses to messages promoting the importance of reducing screen time. As a result of these discussions, the decision was made to reframe the family routine of *restricting screen time* as *promoting free play*, with screen time messages then embedded into the free play component, and also threaded throughout the intervention content.

Responses from the focus groups regarding the delivery format of the program were mixed, with some parents preferring the potential for more tailored information during face-to-face workshops and others preferring the convenience and lower level of commitment required of a Web-based resource. Furthermore, focus group participants revealed that parents would like the workshop delivered by members of the community who were parents themselves and who could report back on their personal experience using the program content. On the basis of this feedback, the decision was made to deliver the program via a workshop and a study website, with the workshop facilitated by a member of the community, rather than a researcher or health professional. Furthermore, given that parents frequently cited lack of time as a barrier to attending workshops, the decision was made to deliver the workshop as an intensive half-day course, rather than requiring parents to commit to several sessions over a number of weeks.

Findings from the initial set of focus groups and parent interviews were then integrated with scientific literature focusing on family routines and rituals [51-55], household chaos [23,56-61], mutually responsive orientation and positive parent-child interactions [47,49,62-64], and developmental science and attachment theory [65-69], and more specifically, how each of these may relate to childhood obesity and weight-related behaviors [23,45,46,58,70-74]. An initial model of the 3PS was then created, which incorporated practical advice for reducing household chaos [75] and promoting positive bidirectional parent-child interactions between young children and their parents [68,76,77]. The 3PS model program was then refined further using an iterative feedback process from 2 end-user panels and 5 focus groups (n=32) with parents. During this process, preferences with respect to the look and feel of the website and study materials, feedback on the program messages and framing, and suggestions for improving the program were explored. In particular, parents reported their partiality for focusing on parent-child relationships as part of the program, which they described as being a neglected aspect of parenting programs to date. The 3PS content to be piloted was then finalized, and the community facilitator who was to deliver the content was trained over 4 days spread over a 4-week period.

Trainings took place at the University and were divided into 4 units. The first unit focused on general skills facilitating groups. During the second unit, the intent, philosophy, and theory behind the 3PS program were delivered. Unit 3 focused primarily on how to deliver the content of the workshop, and unit 4 involved practicing delivery of the content in front of an audience and dealing with any questions or concerns that might arise. The facilitator was also provided with a detailed training manual and access to readings to provide further knowledge and understanding of the program content.

### Intervention

Participants randomized to the intervention group will participate in a half-day, face-to-face workshop and have access to the study website for a period of 6 weeks. The first part of the workshop is theory based and provides parents with insight into their children’s behavior and development [68,76]. It then introduces the Connecting Activities, Routines, and Environments (CARE) framework, which provides a blueprint for introducing routines, while ensuring consideration is given to developing positive bidirectional parent-child interactions and a supportive home environment. The framework is explored in detail, with a particular focus on the importance of family routines [51,53]; positive parent-child interactions [41,43,45,78,79]; and reducing household chaos [56,57,80-82] for supporting child well-being, health, and development. Practical ideas for promoting the parent-child relationship are given [68,76]. These are referred to as *connecting activities*.

A 5-step process for implementing the CARE framework is then introduced to guide parents in finding practical solutions within their own family around getting adequate night-time sleep, participating in family meals, and reducing screen time and promoting free play. In brief, parents identify things they do that might interfere with positive parent-child interactions (step 1). For example, at family meals, a parent using their mobile phone at the dinner table may interfere with positive parent-child interactions. These things are referred to as *disconnecting* activities, and parents are encouraged to identify disconnecting activities that might be modifiable within their own family. Parents then design a simple routine around the activity (step 2) and actively incorporate connecting activities, that is, positive parent-child interactions, into the routine (step 3). An example of bedtime might be a parent tying an imaginary ribbon between the child’s bed and their own bed to *stay connected* through the night. Parents then consider how aspects of the home environment, such as chaos and background distractions, may interfere with the child’s ability to participate fully in the routine (step 4), and then, they are asked to create 1 small, detailed change that they can implement immediately in their home (step 5).

The second part of the workshop is more practical and divided into 3 sections that correspond with the 3 study pillars—supporting sleep, screen-free play, and family meals. Each of the pillars is introduced using a similar format; there is a background reading, which provides a brief overview as to why the activity is important to health and child development, and then the workshop facilitator walks the parents through the 5-step process outlined above as it relates to each specific pillar. For each of the 3 pillars, a number of group activities are used to encourage parent engagement and highlight the study messages and learnings. At the end of the workshop, parents will be given a study pack [15], which includes a meal planner and candle, to promote creating a routine around family meals (eg, lighting a candle at the start of each meal); a child’s book, to encourage reading during the bedtime routine; and a sketch pad and crayons, to encourage free play.

Due to the intensive nature of the workshop, parents will also be given access to a study website. This website will allow parents to return to the material covered during the 3PS workshop and review it at their own pace. In addition to providing all the content delivered during the workshop, the website will also include links to related readings, selected references (as a number of parents said they wanted to know the *science* behind the approach), and extra tips around common feeding and sleep issues in preschoolers. Parents will be sent a link to the study website after the workshop.

Participants log-in to the 3PS website on the study landing page and then are taken to a homepage, which has links to 4 additional pages: (1) the CARE Framework page, which discusses the framework used in detail; (2) the 3 Pillars page, which links to 3 subpages that reflect each of the pillars; (3) an Info page, which provides study-related information; and (4) a Contact Us page, which provides the contact information for the study team. For each of the 3 Pillars subpages, background is provided about how the specific pillar is related to child health and well-being, and a step-by-step approach to using the CARE Framework to address the pillar. For example, with respect to sleep, there is information about what children find disconnecting at bedtime, what a simple bedtime routine might look like, a list of ideas for how parents might be able to infuse connection into the bedtime routine, and then ideas for reducing household chaos to create a peaceful sleep environment. Their access to the study website will be active for a period of 6 weeks.

Participants will be given the choice of attending 1 of the 3 half-day workshops. The day before the workshop, participants will be contacted via email with a reminder of the workshop and also detailed instructions of where to find the room and parking. If a participant does not turn up to the workshop, they will be contacted via phone or email to reschedule the workshop they attend, in an attempt to improve study adherence. The workshop will be catered and dietary requirements provided for.

### Control

Parents allocated to the wait-list control group will not receive the intervention until final follow-up is complete, that is, at the end of the 12-week study period. At this stage, participants will be offered the 3PS program, including the opportunity to participate in the workshop and access to the study website.

### Outcomes

Participants in the intervention group will be asked to complete their 6- and 12-week questionnaires 6 and 12 weeks after the date of the workshop they attend, respectively. Furthermore, 3 workshop dates will be offered on 3 consecutive Saturdays. Participants in the wait-list control group will complete their 6- and 12-week questionnaires 6 and 12 weeks from the date of the first workshop, respectively.

Table 1 illustrates the schedule of outcome assessments for both study groups. All measures will be taken at baseline, 6 weeks, and 12 weeks in both the intervention and control groups, unless stated otherwise. The baseline will contain sociodemographic questions focused on the child, including age, sex, and ethnicity, in addition to questions focused on the caregiver, including age, gender, ethnicity, marital status, employment status, household income, relationship with child, household size, and family structure.

**Table 1:** Schedule of assessments.

Outcomes		Baseline	6 weeks	12 weeks
**Screen use (primary)**				
	Total screen time^a^ (minutes)	x^b^	x	x
	Percentage of children meeting screen recommendations^c^	x	x	x
**Sleep**				
	Brief Screening Questionnaire for Infant Sleep Problems-Extended [83]	x	x	x
**Family meals and nutrition**				
	Frequency of family meals [84]	x	x	x
	Fruit servings per day^a^	x	x	x
	Vegetables servings per day^a^	x	x	x
	Frequency of sugar-sweetened beverages in the previous week^a^	x	x	x
	Frequency of fast foods in the previous week^a^	x	x	x
	Feeding Practices and Structure Questionnaire [85]	x	x	x
**Daily routines and** **household**				
	Child Routine Inventory^d^ [86]	x	x	x
	Chaos, Hubbub, and Order Scale [87]	x	x	x
**Program feedback^e^**				
	Exit questionnaire	—^f^	—	x
	Exit interview	—	—	x

^a^New Zealand Health Survey Questions.

^b^x: measure taken.

^c^Less than 1 hour per day.

^d^Daily Living Routines subscale only.

^e^Intervention group only.

^f^Not applicable.

#### Primary Outcomes

The primary outcome is screen time at 6 weeks, which is one of the program’s targeted routines and the primary endpoint used in a previous study [15]. Furthermore, although increasing screen-free play and, in turn, reducing screen use is one of the targeted routines of the 3PS program, reducing screen use is also promoted as a way to increase connection during family meals and bedtime. For example, parents are advised to turn off screens during family meals and to avoid screen use during the bedtime routine. Given that screen use is either targeted directly or indirectly in each of the 3 routines included in the 3PS program, the decision was made to include it as the primary endpoint for the study.

Screen time will be assessed using 4 questions from the New Zealand Health Survey [88], which were developed by the New Zealand Ministry of Health to measure the amount of time New Zealand children aged 2 to 14 years spend using screens recreationally. Parents report the time (in hours) that their child spends watching television or using *other* screen devices, including mobile phone, tablets, video game consoles, and computers, during weekdays and on weekends. Screen time will be measured as both a continuous variable, that is a decrease from baseline in average screen time, and a binary variable, that is the proportion of children meeting the screen time recommendations of less than 1 hour per day.

#### Secondary Outcomes

Family meals, diet, and parent feeding practices: A single item investigating the frequency of family meals, taken from the validated Family Routines Inventory questionnaire [84] and used in the 45-month *Growing Up in New Zealand* data collection wave, will assess participation in family meals. Moreover, 4 questions from the New Zealand Health Survey will investigate the number of fruit and vegetable servings, fizzy drink consumption, and fast-food consumption. A modified version of the Feeding Practices and Structure Questionnaire [85], which includes 17 questions investigating 4 domains of parental feeding behaviors, will also be administered: (1) reward for behavior, (2) reward for eating, (3) persuasive feeding, and (4) structured meal setting.

Sleep: Sleep will be assessed using the Brief Screening Questionnaire for Infant Sleep Problems-Extended [83] -Adapted. The questionnaire asks parents to report outcomes investigating their child’s nocturnal sleep duration, night waking, method of falling asleep, sleeping arrangements, bedtime rituals, and parental interventions. The Web-based version of the questionnaire has been validated for toddler sleep in a New Zealand and Australian sample [89].

Routines and Home Environment: The *Daily Living Routines* subscale of the Child Routine Inventory [86] will investigate engagement in standard routines of daily life. The subscale involves 11 parent-reported questions. Scores will be summed with higher scores indicating greater frequency of routines. The 15-item Chaos, Hubbub, and Order Scale [87] investigates the level of chaos in the home. A total score will be derived by obtaining a sum of responses for the 15 items, where higher scores represent a more chaotic, disorganized, and hurried home environment.

Exit Questionnaire: The exit questionnaire, completed at 6 weeks by intervention participants only, will assess acceptability and feedback of the 3PS program using 5 open-ended questions about what the participants liked and disliked about the program and what they would keep the same or change.

Exit Interviews: A subgroup of 6 to 10 intervention participants will take part in an exit interview at the 6-week follow-up. The exit interview will be conducted by a trained research assistant on the telephone or face-to-face at a community venue and will take approximately 20 min. Participants will be asked to provide informed consent before participating in the interview. Participants will be prompted to explore in more depth their responses to the exit questionnaire, in particular, whether they used the study website and if they found it helpful. Furthermore, given that the formative work revealed that many parents had concerns about parenting interventions making them feel guilty and judged, we will explore their perceptions of the language and tone used in the program and explore ways in which we could avoid any negative experiences in the future.

### Sample Size

We will aim to recruit 50 people (25 in each group). As this is a pilot trial, it is not powered to detect significant differences between the 2 groups but will provide sufficient data to ascertain recruitment and the direction and likely effect size for the screen-time outcomes.

### Reporting of Results

#### Statistical Analysis

Study data will be collected in REDCap. All statistical analyses will be performed using SAS version 9.4 and will be specified a priori in a statistical analysis plan prepared by the study statistician. All baseline variables will be summarized by group and descriptive summary statistics provided. Analyses will be carried out on an intention-to-treat basis. Chi-square tests, incidence rates, relative risks, and 95% CI will be calculated for all binary variables followed by multiple logistic regression analysis adjusting for other variables if needed. Continuous data will be analyzed using multiple linear regression modeling or nonparametric analysis. Sensitivity analyses will be undertaken to determine the impact of missing data. Repeated measures models will be used to analyze data collected repeatedly over time. A trained research assistant will conduct interviews. Interviews will be recorded (with permission) and transcribed verbatim. A general inductive thematic approach will be followed that allows research findings to emerge from multiple readings of the raw data. NVivo9 software will be used to manage the transcripts and facilitate the analysis process and to identify themes and categories.

## Results

Trial recruitment started in July 2018. Final follow-up was completed in December 2018, with trial findings expected to be available in early 2019.

## Discussion

### Overview

This paper presents the design of an RCT to pilot the effectiveness of the 3PS program. Findings from the pilot study will be used to inform the design of a larger effectiveness study of the 3PS program. Family meals, restricting screen time, and adequate night-time sleep have been shown to promote obesity resilience in young children [13], with evidence that interventions promoting these routines have potential in reducing BMI [15]. At the same time, parents play a central role in instigating and facilitating participation in these daily activities, and as such, promoting positive parent-child interactions and mutually responsive orientation may further foster the development of healthy behaviors with respect to these routines [43,45]. To our knowledge, this is the first reported intervention of a parent-based program that aims to prevent obesity in young children by focusing on positive bidirectional parent-child interactions [47] during the 3 family routines shown to prevent obesity.

### Limitations

This pilot study intervention with short-term follow-up is not able to assess the effect of the 3PS program on child body size. Although the ultimate goal of this approach is to prevent obesity, the decision not to measure children’s body weight was pragmatic, due to limited resources and the short duration of the study. Indeed, although promoting family routines, creating a supportive home environment, and facilitating positive parent-child interactions have the potential to prevent overweight and obesity in later childhood and adulthood, we would not expect to see changes in body weight during the 12-week study period. Finally, although preventing obesity is the long-term aim of this approach, the short-term aims are to improve engagement in the 3 routines shown to protect against obesity, increase engagement in positive parental feeding practices at mealtimes, and reduce household chaos. As such, the study measures have been chosen to reflect these aims.

## Abbreviations

3PS: 3 Pillars Study
BMI: body mass index
CARE: Connecting Activities, Routines, and Environments
RCT: randomized controlled trial
SES: socioeconomic status
